# FROM THE WORKROOM TO THE BEDROOM: MEDIATING EFFECTS OF WORK-TO-FAMILY SPILLOVER ON WORK CHARACTERISTICS AND SLEEP

**DOI:** 10.1093/geroni/igad104.3005

**Published:** 2023-12-21

**Authors:** Kian Huang, Christina Mu, Claire Smith, Soomi Lee

**Affiliations:** University of South Florida, Tampa, Florida, United States; University of South Florida, Tampa, Florida, United States; University of South Florida, Tampa, Florida, United States; The Pennsylvania State University, University Park, Pennsylvania, United States

## Abstract

Negative and positive work experiences may spillover to the home domain and harm one’s sleep. This study investigated whether work-to-family spillover mediates the relationship between work characteristics and sleep health. Full-time workers (n=2,106) from the Midlife in the United States Study provided Time-1 data (T1: 2004-2006); a sub-set (n=1,444) was reassessed at Time-2 (T2: 2013-2015). Both negative work characteristics (perceived unfairness at work and job discrimination) and positive work characteristics (coworker support and supervisor support) were assessed. Mediating variables were negative work-to-family spillover (NWFS) and positive work-to-family spillover (PWFS), and outcome was an overall sleep health score that sums across irregularity, dissatisfaction, nap frequency, inefficiency, and suboptimal sleep duration (higher=poorer sleep health). PROCESS Macro evaluated cross-sectional (T1) and longitudinal (T1-T2) mediation pathways, adjusting for sociodemographics, neuroticism, and work hours. NWFS was a consistent mechanism connecting work characteristics and sleep health. Cross-sectionally, higher NWFS mediated the associations of higher unfairness with poorer sleep health and a sub-dimension of greater dissatisfaction (B=0.09, SE=0.04; B=0.12, SE=0.06, respectively). Higher NFWS also mediated the association between higher discrimination and higher sleep dissatisfaction (p=.008). Longitudinally, an increase in unfairness from T1 to T2 was associated with an increase in irregular sleep at T2 (adjusting for T1 unfairness) through increases in NWFS (p= 0.038). PWFS was not a significant mechanism linking positive work experiences and sleep health, although more coworker and supervisor support were associated with better sleep health. Findings suggest that organizations should limit instances of unfairness and discrimination at work to promote employee sleep health.

